# The Dunedin Multidisciplinary Health and Development Study: overview of the first 40 years, with an eye to the future

**DOI:** 10.1007/s00127-015-1048-8

**Published:** 2015-04-03

**Authors:** Richie Poulton, Terrie E. Moffitt, Phil A. Silva

**Affiliations:** 1 Dunedin Multidisciplinary Health and Development Research Unit, Department of Psychology, University of Otago, Dunedin, New Zealand; 2 Dunedin Multidisciplinary Health and Development Study, Departments of Psychology and Neuroscience, Psychiatry and Behavioral Sciences, Duke University, Durham, NC USA; 3 Social Genetic and Developmental Psychiatry Centre, Institute of Psychiatry, King’s College, London, UK; 4 Dunedin Multidisciplinary Health and Development Study, Christchurch, New Zealand

**Keywords:** Lifecourse, Dunedin study, Research methods, Human development

## Abstract

The Dunedin Multidisciplinary Health and Development Study began more than four decades ago. Unusual at the time, it was founded as a multidisciplinary research enterprise, and was strongly supported by the Dunedin community, both professional and lay, in its early years. Seven research themes have evolved over the past 40 years focusing on mental health and neuro-cognition, cardiovascular risk, respiratory health, oral health, sexual and reproductive health, and psychosocial functioning. A seventh, more applied theme, seeks to maximise the value of the Study findings for New Zealand’s indigenous people—Māori (or tangata whenua transl people of the land). The study has published over 1200 papers and reports to date, with almost 2/3 of these being in peer-reviewed journals. Here we provide an overview of the study, its history, leadership structure, scientific approach, operational foci, and some recent examples of work that illustrate the following: (a) the value of multidisciplinary data; (b) how the study is well positioned to address contemporary issues; and (c) how research can simultaneously address multiple audiences—from researchers and theoreticians to policy makers and practitioners. Near-future research plans are described, and we end by reflecting upon the core aspects of the study that portend future useful contributions.

## A brief history

The Dunedin Multidisciplinary Health and Development Study (hereafter the Dunedin Study) is an ongoing longitudinal investigation of health and behaviour of a complete birth cohort that was drawn from the greater Dunedin metropolitan area (geographical area *n* = 3315 square kilometres, population 120,000), located in the southern coastal region of New Zealand’s South Island. Study participants (*n* = 1037) were born between April 1st 1972 and March 30, 1973 and first followed up at age three, forming the base sample for the longitudinal study. This long-running study was preceded by a pilot study on *n* = 250 four and 5-year-olds born in 1968, led by Phil Silva, a former teacher and educational psychologist (who later became the founding director of the Dunedin study). The pilot sample was drawn from a larger study of perinatal health conducted by paediatrician Dr Patricia Buckfield between 1968 and 1974 [1].

The pilot sought to establish if children who were born in adverse circumstances (e.g. mothers with pre-eclampsia, low birthweight for gestational age, birth trauma) were faring as well as their counterparts who were not born in adverse circumstances. Phil Silva was invited to design and carry out the developmental and psychological assessment in collaboration with personnel from paediatrics, obstetrics, and psychology. This focus on vulnerability around birth and shortly thereafter was partly motivated by the increased sophistication of birth technologies during the 1960s which had resulted in more babies surviving than ever before. A great deal was learned from this early pilot study, not only in terms of the technical and practical aspects of conducting prospective research, but also in terms of substantive findings showing higher than expected rates for some child health problems (e.g. otitis media with effusion a.k.a glue ear), and several publications followed [2, 3].

Because the pilot study was not designed to investigate broader questions of child health and development, and because of a general lack of accurate information in New Zealand about children at that time, it was decided to attempt a larger-scale study of younger children to investigate not only the nature and prevalence of problems of health and development but also some of their correlates, implications, and longer term consequences. The design of what became the Dunedin Study was led by Phil Silva and influenced by longitudinal studies from the UK including the British birth cohort studies [4–6] as well as the Newcastle Upon Tyne Study [7] and the Isle of Wight study [8]. A notable characteristic of these British studies was the inclusion of investigators from a variety of medical and behavioural disciplines, as well as an emphasis on providing practical advice for workers providing services for children and families. Phil Silva sought to establish a similar study in New Zealand.

Given the length of time the study has run, it is perhaps surprising to note that the Dunedin Study was not originally conceived of as a long-term study, particularly as resources were very scarce in the beginning. Indeed, data collection during the early years of the study was helped by contributions from many members of the Dunedin community—doctors who gave freely of their time to conduct medical assessments, volunteers from the wider Dunedin community who helped with other assessments and logistics, schools who gave children a day off to attend the assessments, as well as businesses who allowed parents time off from work to accompany their children to the assessments. A local church even allowed their Sunday school facilities to be used as the study’s assessment space at ages 3, 5, 7, 9, and 11 years.

Without this early community support the Dunedin Study may not have grown to the point where it began to attract the attention of international funding agencies, as well as strengthening the ongoing support from the local New Zealand funders. With regard to the former, some of the first funding from overseas came at the age 13 assessment via a postdoctoral fellowship to Terrie Moffitt (now Associate Director) to support her study of the neuropsychology of juvenile delinquency. It is of passing interest that she employed Richie Poulton, a clinical psychology student at the time and now Director of the Study, as her research assistant to help with these assessments in 1985–86. Since that time the Dunedin Study has enjoyed continuous support from the Health Research Council of New Zealand, plus funding from various branches of the United States National Institutes of Health (e.g., NIMH from 1987; NIDCR from 2003; NIA from 2009) and more recently from the British Medical Research Council (from 2003).

## Leadership structure

The Dunedin Study is led by a Director, an Associate Director, and a team of ‘Theme Leaders’ who share responsibility for the following broad research themes: mental health and cognition, cardiovascular health, respiratory health, sexual and reproductive health, oral health, and psychosocial functioning. A seventh theme explores how the multidisciplinary database can address issues of concern to Māori, New Zealand’s indigenous people. In recent years an Advisory Committee has been established to support the Study director. This governance structure has evolved over the life of the project and reflects a ‘ground up’ process which ensures fitness-for-purpose. The relative simplicity of this structure requires high trust by the host institution, and it has allowed the study to remain focussed on the science, which in turn has allowed it to remain competitive in an unpredictable funding environment. It often surprises people to learn that the Dunedin Study core unit staff have been extramurally funded from its inception until early 2015, when the Director took up a teaching post to strengthen long-term sustainability.

The study’s leadership philosophy has always been straightforward “Treat people as you would like to be treated.” This is a deceptively simple notion. In reality it means devoting a lot of thought to all aspects of what it must be like to be a Study member, most obviously on the day of assessment, but also beyond that, and planning accordingly. In so doing we constantly strive to place ourselves in the Study members’ shoes to better understand their needs and preferences and to accommodate these. For example, knowing that many Study members were parents of young children at the age 32 assessment, we converted our staff tearoom into a comfortable crèche area and employed a part-time professional early childhood care worker to look after Study members children, on site, if this was requested.

## The Study members

The original cohort of 1037 children (52 % males) represents the general population of New Zealand’s South Island in the early 1970s. To be eligible for inclusion participants had to be living in the greater Dunedin Metropolitan area 3 years after their birth at Queen Mary Maternity Hospital—the only maternity hospital in Dunedin at the time. The 9 % who declined or were unable to participate were no different from the 91 % who agreed to take part in terms of maternal prenatal complications, birthweight, neonatal complications or family socioeconomic status [9]. Cohort families represented the full range of socioeconomic status in New Zealand in the early 1970s, as compared to the New Zealand census. Cohort members were primarily white: 7.5 % self-identify as being Māori which matches the ethnic distribution of the South Island of New Zealand. Importantly, we have published evidence that half-a-lifetime of research participation has not improved Study members’ mental or physical health as compared to same-aged participants in the New Zealand National Health and Nutrition Survey’s (e.g. BMI, smoking, visits to the doctor) [10]. Day-long assessments (called phases) have been conducted at ages 3, 5, 7, 9, 11, 13, 15, 18, 21, 26, 32, and most recently at age 38 years (in 2010–2012) when 95 % of the living Study members took part. Our next assessment, at age 45, will begin in 2017.

We have also conducted (or conduct) sub-studies on other generations of the original cohort members’ families. Specifically, interviews with the parents of the Study members took place between 2003 and 2006 for the Dunedin Family Health History Study (FHHS). The FHHS interviews focussed on the physical and emotional health of the extended families (including grandparents and siblings) of the main cohort Study member, with a particular focus on their parents. This study involved 1852 eligible parents of whom 93 % were interviewed, mainly in their own homes. In total they reported on the health status of approximately *n* = 8000 family members [11–13].

We also have an ongoing sub-study of the Study members as parents. As for the FHHS, study data collection occurs in participants’ own homes, around the time of their first child’s third birthday. Of the eligible (actively parenting a 3-year-old child) Study members, 99 % have participated in this study to date and several papers have been published [14–18]. A ‘third’ generation study has recently been added to focus on all children of the Study members when they reach the age of 15 years. These children, and their Study member parent in most cases, are invited back to the Dunedin Study Research Unit where they undergo an assessment similar to that which their parents experienced over a quarter of a century ago, but updated for contemporary issues (e.g., social media use is assessed). A short phone-assessment is conducted with the non-Study member parent at this time, with *n* = 300+ taking part so far.

## Sample retention in the Dunedin Study cohort

As can been seen in table below, all but one of the 12 assessments conducted since birth have enjoyed participation rates well above 90 % (Table 1).

**Table 1:** Retention in the Dunedin Study

Age	Year	Number	Percent^a^ (%)
Birth	1972–1973		
3	1975–1976	1037	100
5	1977–1978	991	96
7	1979–1980	954	92
9	1981–1982	955	92
11	1983–1984	925	90
13	1985–1986	850	82
15	1987–1988	976	95
18	1990–1991	993	97
21	1993–1994	992	97
26	1998–1999	980	96
32	2004–2005	972	96
38	2010–2012	961	95

^a^% of living Study members

Some Study members have missed assessments, but re-enter at later phases.

We work hard to maintain high retention in the Dunedin Study because a major threat to the validity of longitudinal research is non-random attrition. That is, the people who drift away first, or are hard to locate, or hard to re-assess are typically not a random group of participants. Indeed, they tend to be people within whom multiple difficulties aggregate. Thus they must be retained to faithfully capture the full range of life exposures and possible outcomes that occur in the general population. Coming close to this ideal helps strengthen our causal inferences.

**Fig. 1:**
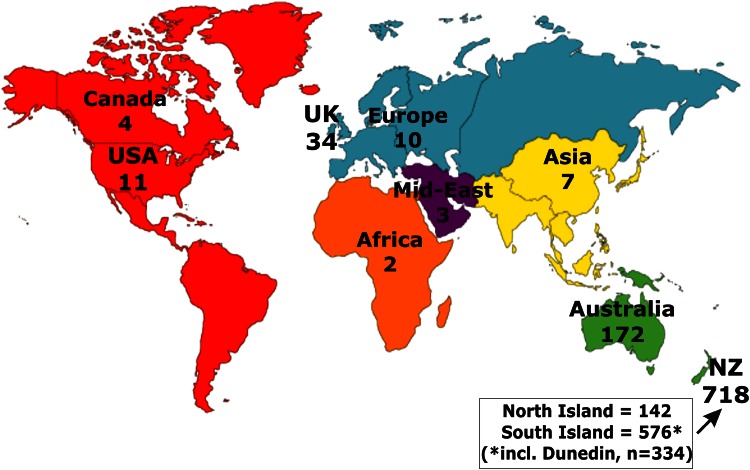
Study member location at age 38

We are often asked about the ‘secret’ to our high retention rates, but there is no secret. In simple terms, it is a product of good systems and careful planning, prioritisation, hard work, and most importantly, the goodwill of our Study members. Initially it involves tracking people who have now moved all over the world, as illustrated in the Fig. 1. This shows Study member locations at our most recent assessment completed in 2012.

As can been seen from the figure, approximately 25 % of the sample currently lived outside New Zealand with only about 30 % of the original sample still living in Dunedin. As one can imagine, there are considerable logistical challenges bringing people back from different parts of the world, accommodating and feeding them whilst in Dunedin (as well as family members on occasion), and getting them home safely. Notwithstanding this dispersion, administrative data matching remains possible in approximately 90 % of the living sample due to high mobility between New Zealand and Australia.

## Key factors in maintaining high participation rates

*Good systems* For maintaining contact and locating Study members, good systems are essential. At each assessment we ask the Study members for contact details of people who know them well and are likely to know their movements over time. We have requested this type of information at multiple phases and have consequently built up a substantive ‘contacts’ database for each Study member. This information is augmented by change-of-address forms returned to us following the mail-out of regular newsletters to Study members. The directors of the study have paid special attention to this task during assessments, helping maintain and strengthen the bond between the study and the Study members over time.

*Good recruitment staff* Location data are used by a ‘sample tracer’ who is employed at each assessment to track, locate, and recruit Study members. A ‘travel co-ordinator’ organises travel plans for the Study member (and other family members if requested). This can be a demanding role, and one we have supported via travel industry training for the staff member concerned.

*Remove barriers to participation* The most obvious barrier is financial; thus wherever possible we provide transportation to and from the Dunedin Research Unit, accommodation, and meals. We also provide a letter to the Study member, addressed to their employer, explaining the value of the study and seeking their co-operation in allowing our Study members to attend the assessment without loss of income. In rare circumstances people who wish to participate but cannot travel to Dunedin (e.g. work commitments, incarceration) are visited by two interviewers in the field (amounting to 1–2 % each phase).

*The assessment needs to be engaging* for Study members and well-balanced across the day to avoid fatigue or boredom. The assessment staff must be well-trained and professional at all times. If one grumpy staff member deals poorly with a Study member, this risks alienating that Study member, and potentially their friends who are also in the study, forever. To ensure staff members remain fresh, week-long breaks are programmed into the assessment phase, and social events occur regularly to keep staff morale high.

*Research outputs* are noticed by Study members. That is, they gain a greater appreciation of the value of their contribution, and they feel respected because the researchers have worked hard and produced good work.

*Appropriate resources* are critical. Good funding is essential. An equally important resource is researcher commitment. Successful longitudinal research takes time. Researchers need to be committed and patient to fully enjoy the fruits of this unusual labour. Also, Study members appreciate continuity in key staff positions, strengthening their ties to the study.

All of these factors are necessary, but not sufficient for high retention. In other words, it is not so much that you do these things (the list above would not surprise anyone); rather, it is how you do these things. This largely comes down to a combination of professionalism, courtesy, and persistence. Paying close attention to the researcher–participant relationship goes a long way to earning Study members’ loyalty.

## Research strategy

The Dunedin Study uses a prospective-longitudinal, correlational design. This enables several different types of studies including: (1) prediction studies of the childhood correlates of later health and behavioural outcomes; (2) developmental studies of onset, course, continuity and change in health and behaviour; (3) epidemiological studies of the prevalence and incidence of health and behaviour problems, and associations among problem types; and (4) methodological studies of reliability and sampling biases.

The basic strategy of the Dunedin Study involves testing for causal relations within this correlational design. We adopt a stepwise approach. First we document that a basic association between two constructs exists, striving for gold-standard (for the time) measurement. Second, we document temporal sequence, as to whether the putative causal variable precedes the outcome variable. Third, we look for a dose–response contingency between the putative cause and outcome. Fourth, we attempt to rule out as many rival causal explanations as possible by introducing control variables into the analyses from our extensive database. Fifth, we test whether putative causal experiences are associated with intra-individual change. Sixth, we try to establish specificity by substituting alternative dependent, then independent variables. Finally, we posit a plausible explanatory process and test this using mediation analyses. By following this general approach we hope to maximise the robustness of our research findings. Ultimately, of course, how ‘true’ a finding is will be determined via independent replication. However, this general approach (augmented by multiple checks including sensitivity analyses) increases the likelihood of successful replication.

While the Dunedin study is correlational (precluding definitive causal statements), there are several features of the study that help strengthen casual inference. These include: (a) a low attrition rate which enhances the accuracy of our effect size estimates; (b) use of multiple data sources—self-reports, tests, observations, records, informant reports (i.e., STORI), which permits replication checks across sources, and allows for construction of multiple-indicator measurement models; (c) our long history of proven confidentiality and non-intervention enhances frank reports on delicate topics; and (d) face-to-face interviewing conducted at the Unit insures privacy which avoids falsification, reduces nay-saying to hasten the assessment process, and facilitates breadth of data collection (e.g. biological data can be collected under controlled conditions, using state-of-the-art technology).

Study members begin their assessment day at 8.45 a.m. and finish around 5.15 p.m. A maximum of *n* = 4 Study members can participate per day and they are run through nine counterbalanced assessment sessions (modules), interspersed by short breaks at morning and afternoon tea, as well as a longer break at lunchtime. We prefer face-to-face interviews because this helps avoid problems with literacy and comprehension (between 5 and 10 % of a general population sample will have at least mild difficulties). We present the assessment modules in a countered-balanced fashion to avoid order effects and to maintain participants’ interest during the 8.5-hour assessment day (e.g. a cognitively taxing assessment is followed by a physiological assessment with low cognitive demand). We hire appropriately qualified interviewers (e.g. a clinical psychologist for the mental health assessment, a dentist for the oral health assessment, and specialist nurses for the cardiovascular assessment) and provide extensive training on the research protocols. Finally, interviewers are kept blind to the Study members’ performance in other sessions. This helps reduce the potential for interview bias and inflated associations from shared method variance.

## The multidisciplinary database

The wide range of data collected over the past four decades, encompassing many aspects of our Study members’ lives, their health, and wellbeing, are displayed in Table 2.

**Table 2:** Archival Dunedin data available for use in longitudinal research

Domains Measured	Ages at which the domain was assessed in the Dunedin Study												
	Birth	3	5	7	9	11	13	15	18	21	26	32	38
Childhood socioeconomic background	×	×	×	×	×	×	×	×					
Academic attainment, literacy tests, degrees earned, skills				×	×	×	×	×	×	×	×	×	×
Work interview: adult socioeconomic status, subjective socioeconomic status, job characteristics										×	×	×	×
Financial interview, saving behaviour, assets, debts, etc.										×	×	×	×
Upbringing (many different measures)													
Parental mental health		×	×	×	×	×	×	×					
Family structure	×	×	×	×	×	×	×	×					
Family functioning, parent–child relationship		×	×	×	×	×	×	×					
Parental loss, familial death, divorce		×	×	×	×	×	×	×			×		×
Maltreatment (many caregiver changes, sexual abuse, physical abuse, harsh discipline, mother–child observations by staff)		×	×	×	×	×	×	×					
Retrospectively recalled child abuse											×		×
Peers, rejection by, attachment to, activities with			×	×	×	×	×	×	×	×	×	×	×
Adult relationships (quality, commitment), family formation										×	×	×	×
Intimate partner abuse (psychological, physical, injury)										×	×	×	×
Child temperament, via staff observations		×	×	×	×	×							
Personality traits, MPQ and big-5 (self, staff, and informant)									×		×		×
Antisocial behaviours (self-reports, parent & teacher reports, informant reports, police records)			×	×	×	×	×	×	×	×	×	×	×
Mental disorders and psychopathology (parent, teacher, self, and informant reports, medical records)			×	×	×	×	×	×	×	×	×	×	×
Substance abuse and dependence (parent, teacher, self, and informant reports)						×	×	×	×	×	×	×	×
Health-compromising behaviours													
Smoking, alcohol, cannabis, hard drugs					×	×	×	×	×	×	×	×	×
Risky sexual behaviour, dangerous driving									×	×	×	×	×
Self-harm, suicide attempts								×	×	×	×	×	×
Paediatric neurological examinations and motor tests		×	×	×	×								
Neuropsychological testing, IQ testing, language		×	×	×	×	×	×						×
Foetal/newborn perinatal health (hospital records) and direct observations	×												
Ratings of child’s health, ill-health checklists		×	×	×	×	×	×	×					
Pubertal timing (girls’ menarche, boys’ height spurt)					×	×	×	×	×				
Health Status (many different measures)													
Respiratory: asthma, allergy, and lung function tests					×	×	×	×	×	×	×	×	×
Dental examinations: periodontal disease, caries			×		×	×	×	×	×	×	×	×	×
Reproductive: STDs, sexuality, pregnancy history									×	×	×	×	×
Interviews: headache, pain, sleep, self-reported health										×	×	×	×
Injuries		×	×	×	×	×	×	×	×	×	×	×	×
Fitness tests													
Cardiorespiratory Fitness (VO_2_ max; maximal aerobic capacity)								×			×	×	×
Grip strength													×
Balance													×
Physical exercise/activity				×	×	×	×	×	×	×	×	×	×
Diet											×	×	×
Biomarkers													
Blood pressure, heart rate		×	×	×	×	×	×	×	×	×	×	×	×
Adiposity (including body composition), anthropometrics	×	×	×	×	×	×	×	×	×	×	×	×	×
*Biomarker Assays*, e.g. full blood count, cortisol, testosterone, lipids, inflammation (hsCRP, Fibrinogen, IL-6)											×	×	×
Digital imaging of retinal micro-vasculature													×
Endothelial function													×
*DNA/RNA bank*, from blood,											×		×
Genome-wide SNP scan													×
Leukocyte Telomere length											×		×
*Family history* of psychiatric and medical illnesses, from interviews with three informants per family												×	
Teacher/School reports			×	×	×	×	×	×					
*I* *nformant reports* of financial, social, physical and mental health, cognitive status, and personality traits.										×	×	×	×
*Administrative records:* national health records, pharmacy prescriptions, accident/injury compensation records, social welfare benefit records, VEDA credit Ratings, court conviction records										×	×	×	×
*Home*-*visit parenting assessment* of Study members with 3-year-old child									×	×	×	×	×
*Next generation assessment* of Study members’ 15-year-old													

## The findings

To date there have been over 1200 publications and reports from the Dunedin Study, with approximately two-thirds of these appearing in peer-reviewed journals. In what follows, we have selected some recent papers from the Dunedin Study that illustrate six important features of our research: (a) the value of multidisciplinary data; (b) the importance of programmatic research insomuch as a number of these recent papers build on earlier work published from the study; (c) how our research programme tackles both specific contemporary questions with high translational value whilst also addressing some more timeless ‘big science’ issues (e.g. nature-nurture interplay, the psyche-soma interface); (d) our attempts to speak simultaneously to multiple audiences by drawing out implications for theory, research, practice, and policy; (e) our strong emphasis on making sure the research is (re) packaged in such a way to ensure accessibility and wide impact; and (f) ‘niche sensitivity’ as we try to play to the Dunedin Study’s strengths (i.e., half a lifetimes detailed socio-behavioural data allowing for a holistic view of physical health and wellbeing). We end with some illustrative examples of how the researchers ‘follow through’ to maximise the uptake and utility of the research.

*Example 1: Self-control in childhood is more important than socioeconomic status (SES) or IQ in predicting adults’ physical health, wealth, life satisfaction, addiction, crime, and parenting of the next generation* (see [19–22]). One project that has attracted considerable attention from colleagues, policy makers, the media, and the public is about the importance of self-control skills mastered in childhood for success in all facets of adult life. The findings have been interpreted as lending support to the growing movement for quality early-childhood education.

This work is the logical extension of an ongoing 20-year programme of research focused on temperament and personality continuities and life consequences (e.g., [23–27]).

*Example 2. Measuring early conscientiousness can identify which now-healthy patients will develop health problems in the future* [28]. This line of research brings translational medicine and personality psychology together. Our translational research suggests that a 5-item questionnaire given in the waiting room at the GP’s office can tell doctors which patients need more motivational counselling to promote healthy behaviours and prevent later onset of disease. The latest findings are the logical extension of a 15-year research programme examining if and how behavioural style predicts poor health (e.g., [29, 30]).

*Example 3: Long-term cannabis users at midlife* In 2002 we reported that young cannabis users had elevated risk for psychosis [31]. In 2007 we reported that cannabis users developed periodontal disease, beyond the effects of tobacco use [32]. Our Dunedin Study is well suited to tracking the health of sustained heavy cannabis users as they age. In 2012 there was strong interest in our report in PNAS [33, 34] that cannabis users who began using as teens and continued into adulthood showed a variety of cognitive declines, culminating in a loss of 8 IQ points on formal testing from age 11 to age 38. In January 2013, this paper was again prominent as result of a misguided critique attributing our findings to socioeconomic factors [35] to which we published a successful empirical rebuttal [36]. In January 2014, this paper was once again in the news, as result of legalisation of cannabis in Colorado, and because it was featured in an AMA report that recommended against legalisation. Lost in the hyperbole was our observation that evidence of harm is clearest for daily cannabis smokers, but we did not detect deleterious effects in less regular users. The Dunedin Study also has new findings about other risks (and non-risks) to long-term cannabis users [37, 38].

This work follows earlier papers going back as far as the mid-nineties documenting widespread cannabis use among young New Zealanders and the policy implications of such (e.g., [39–42]).

*Example 4: Psychosocial distress is associated with accelerated telomere erosion.* We have measured telomere length in the Dunedin cohort at ages 26 and 38 years, allowing us to be one of the first research teams to test telomere erosion over time (not just length at one point in time). In our study, erosion was accelerated among cohort members who experienced psychosocial distress [43]. These findings will help to unravel how early experiences affect individual differences in the pace of ageing and age-related disease [44], including among those experiencing persistent asthma since childhood [45]. The latter finding builds on earlier work describing the natural history of, and risk factors for, asthma and atopy from childhood to adulthood (e.g., [46, 47]).

*Example 5: Documenting how Genome Wide Association Study (GWAS)-discovered genetic risk shapes the development of illness and health*. GWAS are turning up “hits” for many diseases, and the next step is to uncover how these genetic variants work. Some labs take the bottom-up approach, tracing pathways of gene function from the DNA up through proteins and cells, toward disease. In contrast, we take the “top-down” approach, asking how genetic variants are connected to key clinical features of the disease itself, and how disease unfolds across the life-course.

We have a new series of publications reporting discoveries about three GWAS success stories: obesity, smoking, and asthma. These three conditions are known to accelerate physical ageing. Because we have traced these phenotypes in the Dunedin Study as they unfold from birth to midlife, we were ideally situated to ask the following: exactly when in the course of human development does genomic risk ascertained in GWAS hits first come on line? We reported that genes detected in GWAS of obese adults are unrelated to birth weight, but they influence rapid growth from birth to age 3. The link from genes to adult obesity is accounted for by this rapid infant growth [48, 49]. We found that genes detected in GWAS of adult smokers are unrelated to cigarette initiation, but they influence rapid progression from first cigarette to addiction. The link from genes to failure of adult smoking cessation is explained by this initial fast progression in teenagers. We further found that ‘chippers’ who can smoke without becoming addicted carry less-than-normal genetic risk [50]. In asthma, genes detected in GWAS of asthmatic adults are linked specifically to childhood-onset persistent course, and to a variety of respiratory phenotypes indicating extreme severity and chronicity of the disease [51, 52].

These findings tell GWAS teams which phenotypes to test to find more genes: early onset plus persistent course. They also indicate that early life is a good time to prevent the effects of genetic risks on lifelong health and ageing. Finally, we found that genomic risk scores are surprisingly un-correlated with family history of each disease. This suggests that requiring family history as a gate for genetic diagnostics may be misguided for common disorders.

This work builds on a stream research that began more than a decade ago in which the Dunedin Study provided the first clear demonstration of gene-environment interaction in the behavioural sciences using measured genes and measured environments [53]. This was followed by series of papers modelling nature–nurture interplay in the behavioural [54–56]; neuro-cognitive [57] and respiratory domains [58]. This type of research illustrates the value of having a bio-repository with DNA, as well as high-quality repeated measures of environmental exposures from birth onwards. These studies have helped stimulate interest, particularly among behavioural scientists, in gene–environmemnt interaction (GxE). One of the key messages from this work is that genes by themselves tell us very little; it is the combination of genes and environments that matters most. The implication is that attempts to modify the environment remain a very sensible intervention strategy for improving health and development.

*Example 6: Evidence of neurodegeneration in schizophrenia, detected through mental testing and digital imaging of the retina*. In a series of publications we have tracked members of the Dunedin birth cohort who were diagnosed with schizophrenia. We reported that different cognitive functions show change on different trajectories from childhood to adolescence and forward, past disease onset into midlife. The key deficit is in “processing speed”. It lags already in early childhood for individuals who will later develop schizophrenia, plus the deterioration of processing speed accelerates even more after schizophrenia diagnosis [59–61]. We further showed the benefits of applying digital retinal imaging technology in the study of schizophrenia, as the retina is a non-invasive window on the condition of the vasculature inside the brain and body. Through retinal imaging, we discovered expanded venular calibre is characteristic of schizophrenia, but not other related physical or mental disorders [62, 63]. This finding made for a captivating cover photo of a human retina that appeared on the cover of the December 2013 edition of the *American Journal of Psychiatry.* It has since been replicated in Australia. Our discovery shows that retinal imaging is an inexpensive non-invasive technology that can be used to track changes in brain integrity and cognitive decline over the life-course, also see [64].

*Example 7: Effects of prospectively ascertained early-life adversity on adult physiology and physical health in midlife*. The Dunedin Study team has continued its investigations into how early-life psychosocial stress is converted to physiological abnormalities in biomarkers, thus leading to poor health and accelerated ageing. This work began in earnest 10+ years ago by showing that growing out of childhood socioeconomic disadvantage by adulthood did not undo or mitigate the damage caused by early adversity [65]. This was followed by data showing elevated inflammation via C-reactive protein, fibrinogen, and white blood cell count in adult victims of child abuse [66, 67]. We brought together multiple childhood risks (socioeconomic disadvantage, social isolation, maltreatment) and multiple age-related disease entities (depression, inflammation, cardiovascular risk) to show prediction beyond traditional risk factors, and critically, the need for a multi-pronged intervention policy response because of non-redundancy among the childhood risk factors [68].

*Example 8: The very high cumulative prevalence of psychiatric conditions over the life course.* We reported that the prevalence of anxiety, depression, and substance dependence is about twice as high as the mental health community has been led to believe. It depends on how you measure mental disorder. By using the long-term Dunedin Study tracking a thousand New Zealanders from birth to early midlife we showed that people significantly underreport the amount of mental illness they have suffered when they are asked to recall their history in diagnostic interviews years after the fact [69, 70]. Since 2010, our initial finding has been replicated by four other cohort studies (in Oregon, Minnesota, North Carolina, and Baltimore). As a result of this provocative new finding, researchers are beginning to ask why so many people experience a disorder at least once during their lifetimes and what this means for the way we define mental health, deliver psychiatric services, and count the economic burdens of mental illness [71, 72]. At the very least, the finding that most of us will experience an episode of mental disorder if we live long enough should help reduce the stigma against mental illness.

*Example 9: Young suicide attempters need follow-up care after emergency-room treatment*. The rate of suicide attempts is rising in today’s struggling economy, and the majority of attempters survive, raising the question of what will happen to them. Service delivery studies reveal that most attempters receive no further care after emergency-room treatment of their injury. Our Dunedin cohort came of age in a deep economic recession in New Zealand in the 1990s, giving us the opportunity to ask what happened to suicide attempters when followed up 15–20 years later, to midlife. We compared them to controls matched on psychiatric history, to highlight whether their suicide attempt signalled more prognosis than clinicians could glean from their initial psychiatric diagnoses. The life outcomes of attempters were very poor across the board, including poor physical health and financial dependency. Particularly concerning was that they were harming others, with high rates of violent crime, intimate partner violence, and having their children go into care for child protection [73].

*Example 10: Psychotic symptoms in young children: what do they mean?* In 2000, Dunedin Study researchers were the first to report that children as young as age 11 experience delusions and hallucinations [74]. We found that although such symptoms are rare, those who report them have strongly elevated risk for diagnosis with a psychotic illness when they reach adulthood. This Dunedin Study finding has been replicated in over a dozen studies since 2000. Our more recent findings are that children who experience such symptoms tend to have the same neuropsychological and psychosocial characteristics as adults with schizophrenia [75] and that they exhibit high rates of comorbid disorders in addition to psychosis when they reach midlife [76]. These findings underscore that the development of poor mental health is a lifelong process. Because people with serious mental illness are at risk for early morbidity and mortality, these findings suggest that the early years of life represent a propitious opportunity for intervention to enhance late-life quality of life. The possibility of preventing or even pre-empting the negative consequences of childhood psychotic symptoms spurred us to partner with researchers running intervention studies. Initial findings are promising [77].

## Impact beyond the academy

From the earliest days, the Dunedin Study has had societal impact (e.g., [78–82]). This can be seen initially in the 1982 report “Child Health and Child Health Services in New Zealand”. This was a landmark document at the time and was heavily informed by findings from the first decade of the Dunedin Study, both in terms of mapping the prevalence of child health problems, and informing resource allocation and service planning for New Zealand children by the Ministry of Health. Indeed, a nation-wide health and development screening programme grew out of this work and was an important precursor to the New Zealand Ministry of Health’s Well-Child programme currently administered by the Plunket Society (a national baby and infant home visiting service founded 107 years ago), employing specialist nurses for seven mandated health checks between birth and age 5, and available to all comers). The ‘Health and Development Record’ followed, which for the first time held all the health information on a child in one place from birth through to adolescence. It was designed with charts and notes to be updated by nurses, doctors, teachers, and parents as children developed. It was accessible and understandable to people from all walks of life. As well as spaces to record development, immunisation, height and weight, eyes and ears, and behaviour, there were general tips and guidelines for parents. The booklet also provided developmental information on what parents might expect their children to be doing from birth through to primary school and gave advice on what to do in case of accidents or illnesses.

Equally important at the time, injury research by John Langley and colleagues was instrumental in ensuring thermostats were introduced into hot water cylinders in New Zealand to reduce the risk of scalds and burns among children [83–88]. His group also highlighted the importance of practical injury prevention strategies in the playground [84, 89–93]. The ubiquitous safety mats seen under playground equipment today resulted from early work and assiduous advocacy by the Dunedin Study injury researchers. Even the length of electric jug cords, with longer leads providing greater opportunity for child injury were investigated, resulting in real-world changes reducing innumerable scalds and accidents in the kitchen, via reduction in the length of the cords [94].

By the 2nd decade of the study, foundations were being laid for work that ultimately distinguished between children with early-onset and persistent antisocial behaviour versus those whose antisocial behaviour began in adolescence. These two groups have different aetiologies and require different policy responses. This distinction has now been enshrined in diagnostic taxonomies, and according to the Principal Youth Court Judge (Andrew Beecroft), has profoundly influenced judicial practice in New Zealand. Further afield, this work contributed to the *Amicus Brief* that led to the overturning of the death penalty for youth under the age of 18 in the United States of America. The impact and significance of this research were recognised in 2007 when Terrie Moffitt was awarded the Stockholm Prize.

In the past decade, the Dunedin Study has taken the lead internationally in modelling how nature (our genes) interacts with nurture (our life experiences) to help predict why people behave the way they do. For example, we have shown that among children who were maltreated, those that go on to repeat the cycle of violence have a specific form of the MAOA gene, whereas those with a different form of this gene appeared resilient to maltreatment. We have also shown that the likelihood of developing psychosis in adulthood after exposure to cannabis use in adolescence is conditional, that is, it depends upon the presence of a particular ‘vulnerability’ genotype. In perhaps the best known of these nature–nurture interplay studies, we showed why certain people succumb to depression in the face of life stress, whereas others do not. This finding, along with several others, was voted to be the second most important scientific breakthrough in the world—in any branch of science—in 2003.

Together, these studies about nature and nurture interplay and behaviour have led to a paradigm shift in the way people think about the old nature *versus* nurture debate. In essence, this notion of one *or* the other is moribund. As popularised by Matt Ridley in his book, “it’s more a case of “Nature via Nurture” [95]. The impact of these findings stretches well beyond the academy and the popular press. For example, several years ago, an Italian judge overturned a sentence based on the argument that the MAOA gene conferred susceptibility to violence, and this could be used in mitigation at sentencing. Putting aside the rights and wrongs of this decision, it is also clear that the basic associations reported by the Dunedin Study at the population level have spurred a great deal of neuroscience work in the laboratory, trying to uncover the mechanisms that might be involved.

Reflecting its multidisciplinary character, the Dunedin Study is the only study in the world to have collected data on the same individuals’ oral health from age 5 continuously through to mid-life. Capitalising on this unique data, Murray Thomson led a study that appeared in the Journal of the American Medical Association (JAMA) identifying cannabis use as a novel risk factor for periodontal disease [96]. At the time, it was one of only two oral health research papers to have appeared in this prestigious general medical journal in the previous 20 years. Another, more recent oral health paper addressed the fluoride-IQ debate, generating a deal of interest among researchers and the wider community [97].

Data on substance use/dependence, mental health and from the sexual health theme led by Nigel Dickson (e.g. [98–104]); also see [105, 106]) have been used in a variety of New Zealand policy-making contexts (e.g. Health Select Committee reports, Law Commission reports, and a variety of professional bodies, e.g. the NZ Herpes Foundation), as well as overseas (e.g. WHO, UK House of Lords, the U.S. Presidential Office, US Surgeon-General’s reports). One recent example of impact in the U.S. context relates to a series of reports produced by the Center on the Developing Child at Harvard University and the American Academy of Pediatrics about the ‘new science’ of child development in which research from the Dunedin Study features [107, 108].

## Making the findings matter

Demonstrating utility is increasingly expected by public-good research funders. Being able to provide concrete evidence of impact on policy and/or practice enhances the likelihood of continued support, plus evidence of impact is good to feed back to Study members. This suggests that longitudinal researchers need to engage more directly with policy makers. This can be challenging insomuch as policy-makers typically respond to different contingencies than do researchers, and the demands of the policy world (e.g. time-sensitive need) can feel antithetical to carefully conducted, rigorous research.

As the foregoing suggests, the founding Dunedin Study Director Phil Silva was very effective in providing policy advice to multiple New Zealand government agencies and the main political parties during the first decades of the study. The current zeitgeist values such activities and hence they are prioritised by the current Director Richie Poulton. Some examples of recent translational efforts include his membership on the multi-agency (Ministries of Health, Education, Social Development) Advisory Group on Conduct Problems between 2007 and 2012 [109–112], the Prime Minister’s Advisory Group on Reducing the Social and Psychological Morbidity in the Transition through Adolescence in 2010–2011 [113], the National Government’s Early Childhood Education (ECE) Taskforce in 2011 [114], and the Children’s Commissioner’s Expert Advisory Group on Solutions to Child Poverty in 2012 [115, 116]. Further expanding translational opportunities, he has recently been appointed to a part-time role as the inaugural Chief Science Advisor to the Ministry of Social Development, New Zealand’s largest ‘social good’ ministry.

The Study’s Associate Director, Terrie Moffitt, is based in the USA and also takes on many advisory roles including the American Psychiatric Association’s DSM-V, The U.S. National Academy of Sciences, The Nuffield Foundation (UK), the Jacobs Foundation (Switzerland), and the Trygfonden Child Intervention Research Center (Denmark).

## The future

The Dunedin Study will keep pushing the scientific envelope. For example, we are currently planning to conduct a brain imaging study of our Study members in their mid-40s. The fMRI study will collect structural and functional neuroimaging measures to address three main aims. First we plan to test the hypothesis that early-life adversity predicts variation in the function, structure, and connectivity of four neural hubs and their core behavioural capacities. Hubs include: (1) the amygdala and emotion/threat; (2) the ventral striatum and motivation/reward; (3) the hippocampus and memory; and (4) the dorsolateral prefrontal cortex and executive control. We will focus on prospectively ascertained adversities that predict disease morbidity and mortality, specifically childhood socio-economic deprivation, social isolation, and child maltreatment.

Second, we plan to test the hypothesis that individual differences in the function, structure, and connectivity of these same four neural hubs are correlated with real-world behaviours needed to prepare for successful ageing. We will focus on neural correlates of midlife real-world behaviours outside the lab (i.e. “neuroecology”) that prepare individuals for health and wellbeing in late life, such as preparing for the financial future, building social relationships, and sustaining positive emotions.

Third, we will test the hypothesis that individual differences in the function, structure, and connectivity of these four neural hubs are correlated with the pace of biological ageing. The Dunedin Study has comprehensive biomarkers of metabolic, liver, kidney, cardiovascular, dental, immune, and respiratory function, facial ageing, and telomere length measured repeatedly at ages 26, 32, and 38 years. With another assessment at age 45 (see more below) we will be able to reliably estimate slopes of decline in each biomarker. Modelling correlated “slopes” of decline among the biomarkers as cohort members enter midlife will allow us to quantify each individual’s pace of ageing and correlate this pace with neural measures.

Our planned age 45 assessment will begin in 2017 and finish toward the end of 2018. We plan to gather new data to augment the extensive data already held. We will test a number of hypotheses aimed at better understanding the complex process of ageing. We will also expand our data collection efforts to capitalise on data already held about conditions associated with very high rates of disability [117], e.g. musculoskeletal problems and chronic kidney disease.

Data linkage of national administrative databases has recently become more feasible in New Zealand and we are working hard to take advantage of the opportunities this provides, of course, with the appropriation permissions from our Study members.

Links to other studies are more important than ever. One of the most critical needs in behavioural and health sciences is replication of findings across different nations and population groups [118, 119]. We must document the robustness of each finding, and we must also delimit when and where it does not apply. Thus, communication among research teams and harmonisation of data collection and analyses are necessary if we are to translate findings into improved health care. Here we have described the Dunedin Study, and we are currently working to consolidate links with other similar studies, for example, the Christchurch Health and Development and Study in New Zealand and the E-Risk Study in the UK.

## But remember to do the basics right…

As noted by McDermott [120] one of the hallmarks of the Dunedin Study has been the ability to horizon scan effectively and respond wisely. In a rapidly morphing scientific landscape, this will be more challenging than ever. It means reading widely, broadening the range of researchers and skill-sets that make up the Dunedin Study team, and linking with and learning from others. Notwithstanding, the key characteristics that have made the Dunedin Study successful to date—respect for our Study members, our multidisciplinarity, our high retention rate, our ‘deep’ measurement, and our clear policies guiding scientific governance and ethics—will continue to be the bedrock upon which our future scientific contributions rest.
